# The baseline hemoglobin level is a positive biomarker for immunotherapy response and can improve the predictability of tumor mutation burden for immunotherapy response in cancer

**DOI:** 10.3389/fphar.2024.1456833

**Published:** 2024-10-02

**Authors:** Yin He, Tong Ren, Chengfei Ji, Li Zhao, Xiaosheng Wang

**Affiliations:** ^1^ Biomedical Informatics Research Lab, School of Basic Medicine and Clinical Pharmacy, China Pharmaceutical University, Nanjing, China; ^2^ Big Data Research Institute, China Pharmaceutical University, Nanjing, China; ^3^ Cancer Institute, Xuzhou Medical University, Xuzhou, China; ^4^ Center of Clinical Oncology, The Affiliated Hospital of Xuzhou Medical University, Xuzhou, China; ^5^ Jiangsu Center for the Collaboration and Innovation of Cancer Biotherapy, Xuzhou Medical University, Xuzhou, China; ^6^ Beijing Highthink Pharmaceutical Technology Service Co., Ltd., Beijing, China; ^7^ Public Experimental Platform, China Pharmaceutical University, Nanjing, China

**Keywords:** pan-cancer, hemoglobin level, immune checkpoint blockade, immunotherapy response, predictive biomarker

## Abstract

**Purpose:**

Because only a subset of cancer patients can benefit from immunotherapy, identifying predictive biomarkers of ICI therapy response is of utmost importance.

**Methods:**

We analyzed the association between hemoglobin (HGB) levels and clinical outcomes in 1,479 ICIs-treated patients across 16 cancer types. We explored the dose-dependent associations between HGB levels and survival and immunotherapy response using the spline-based cox regression analysis. Furthermore, we investigated the associations across subgroups of patients with different clinicopathological characteristics, treatment programs and cancer types using the bootstrap resampling method.

**Results:**

HGB levels correlated positively with clinical outcomes in cancer patients receiving immunotherapy but not in those without immunotherapy. Moreover, this association was independent of other clinicopathological characteristics (such as sex, age, tumor stage and tumor mutation burden (TMB)), treatment program and cancer type. Also, this association was independent of the established biomarkers of immunotherapy response, including TMB, PD-L1 expression and microsatellite instability. The combination of TMB and HGB level are more powerful in predicting immunotherapy response than TMB alone. Multi-omics analysis showed that HGB levels correlated positively with antitumor immune signatures and negatively with tumor properties directing antitumor immunosuppression, such as homologous recombination defect, stemness and intratumor heterogeneity.

**Conclusion:**

The HGB measure has the potential clinical value as a novel biomarker of immunotherapy response that is easily accessible from clinically routine examination. The combination of TMB and HGB measures have better predictive performance for immunotherapy response than TMB.

## Introduction

Immune checkpoint blockade (ICB), which reinvigorates antitumor immune responses by interrupting co-inhibitory proteins in PD-1/PD-L1/CTLA-4-related pathways, have gained great success in treating various cancers (Liebl and Hofmann, 2019; Darvin et al., 2018). However, only subset (<20%) of cancer patients can benefit from ICB therapies to date (Paucek et al., 2019), highlighting the pressing need to develop reliable predictive biomarkers for the response to ICB. Although some such biomarkers have been established, such as tumor mutation burden (TMB), microsatellite instability (MSI) or mismatch-repair deficiency (Le et al., 2015), and PD-L1 expression (Khunger et al., 2017), oncologists are frustrated by the lack of high predictive accuracy of these biomarkers. In addition, although some potential biomarkers for immunotherapy responses have been investigated, such as T cell infiltrate (Bruni et al., 2020), tumor aneuploidy (Davoli et al., 2017) and mutations in specific genes (Li et al., 2020a; Shen et al., 2018), the clinical utility of these biomarkers remains unclear.

Hemoglobin (HGB) is the protein contained in red blood cells for delivery of oxygen to the tissues to ensure adequate tissue oxygenation. Several studies have explored the association between the HGB level and clinical outcomes in cancer patients treated with immune checkpoint inhibitors (ICIs). For example, Gou et al. (2021) investigated the link between pretreatment HGB levels and outcomes in 197 advanced or metastatic gastric cancer patients receiving anti-PD-1 antibody treatment. This investigation revealed that the patients with normal HGB levels (≥110 g/L) had prolonged overall survival (OS) and progression-free survival (PFS) compared to the patients with decreased HGB levels (<110 g/L). However, both groups of patients showed no significant difference in the objective response rate (ORR) (Gou et al., 2021). Another study by Zhang et al. (2020) analyzed 310 late-stage non-small cell lung cancer (NSCLC) patients receiving ICI therapies and found that the patients with normal HGB levels (≥110 g/L) had significantly longer OS and PFS than those with lower HGB levels. Concordantly, these studies have shown a positive association between HGB levels and survival prognosis in cancer patients in the context of immunotherapy. Unfortunately, these studies showed no evidence that the association between HGB levels and survival is a consequence of immunotherapy response in cancer patients. As a result, whether HGB levels indeed have a positive association with immunotherapy response remains unclear. To this end, this study aimed to address several key issues concerning the association between the HGB level and immunotherapy response. First of all, is the association significant and positive in pan-cancer? Second, is the association confounded by other factors, such as age (Cappellini and Motta, 2015), sex (Murphy, 2014) and treatment program (Pirker et al., 2013)?

To address these issues, in this study, we analyzed the association between HGB levels and clinical outcomes in 1,479 ICIs-treated patients across 16 cancer types. We explored the dose-dependent associations between HGB levels and OS, PFS and immunotherapy response using the spline-based cox regression analysis. Furthermore, we investigated the associations across subgroups of patients with different clinicopathological characteristics, treatment programs and cancer types with the bootstrap resampling method. In addition, we explored the associations of HGB-related genes expressions with phenotypic and immune-relevant molecular features in two pan-cancer cohorts, including TCGA (https://portal.gdc.cancer.gov/) and Pancancer_2020 corhorts (Consortium et al., 2020), who were not treated with immunotherapy.

## Methods

### Data collection

We accessed publicly available data of 1,479 cancer patients across 16 cancer types from Memorial Sloan Kettering Cancer Center (MSKCC) (Chowell et al., 2022). These patients were treated with PD-1/PD-L1 and/or CTLA-4 antibodies. Their HGB levels were assessed before ICB treatments. We downloaded multi-omics and clinical data for 32 TCGA cancer types and TCGA pan-cancer cohort from UCSC Xena (https://xenabrowser.net/datapages/). The 32 cancer types included adrenocortical carcinoma (ACC), bladder urothelial carcinoma (BLCA), breast invasive carcinoma (BRCA), cervical squamous cell carcinoma and endocervical adenocarcinoma (CESC), cholangiocarcinoma (CHOL), colon adenocarcinoma (COAD), lymphoid neoplasm diffuse large B-cell lymphoma (DLBC), esophageal carcinoma (ESCA), glioblastoma (GBM), head and neck squamous cell carcinoma (HNSC), kidney chromophobe (KICH), kidney clear cell carcinoma (KIRC), kidney papillary cell carcinoma (KIRP), acute myeloid leukemia (LAML), brain lower grade glioma (LGG), liver hepatocellular carcinoma (LIHC), lung adenocarcinoma (LUAD), lung squamous cell carcinoma (LUSC), mesothelioma (MESO), ovarian serous cystadenocarcinoma (OV), pancreatic adenocarcinoma (PAAD), pheochromocytoma and paraganglioma (PCPG), prostate adenocarcinoma (PRAD), rectum adenocarcinoma (READ), sarcoma (SARC), skin melanoma (SKCM), stomach adenocarcinoma (STAD), testicular germ cell tumors (TGCT), thyroid carcinoma (THCA), thymoma (THYM), uterine corpus endometrial carcinoma (UCEC), and uterine carcinosarcoma (UCS). In addition, we downloaded a pan-cancer dataset (Pancancer_2020) which included 20 cancer types from cBioPortal (https://www.cbioportal.org/study/summary?id=pancan_pcawg_2020). A summary of these datasets is shown in Supplementary Table S1.

### Exploring the dose-dependent relationship between HGB levels and survival prognosis

To uncover the non-linear relationship between HGB levels and OS and PFS in cancer patients, we introduced restricted cubic splines estimation into univariate and multivariate Cox proportional hazards regression models. The multivariate models included the covariates of sex, age, body mass index (BMI), tumor stage, chemotherapy, ICI therapy method and TMB. The “cph()” and “rcs()” functions in the “rms” R package (version 6.7.0) were used to construct the regression models. By taking the median value of HGB levels (117 g/L) as a reference, hazard ratios (HR) were estimated across the continuous spectrum of HGB levels. We employed the “predict()” function in “rms” to calculate the HR with 95% confidence intervals (CIs). Similarly, we used univariate and multivariate Cox regression analyses to estimate the prognostic values of different baseline HGB levels in cancer patients.

### Survival analysis

We conducted survival analyses using the “survfit()” function in the R package “survival” (version 3.5.5). Kaplan-Meier curves were utilized to display the OS and PFS time differences between different groups of cancer patients. The log-rank test was used to assess the significance of survival time differences. We executed the function “tbl_survfit()” in the R package “survminer” (version 0.4.9) to calculate the median survival time of OS and PFS with the parameter “probs = 0.5”.

### Bootstrap resampling

We used the R function “sample_n()” with the parameter “replace = TRUE” to generate 1,000 randomly resampled cohorts. For each resampled cohort, we performed corresponding statistical analyses.

### Logistic regression analysis

We randomly divided the patients into training (70%) and test sets (30%) using the “createDataPartition()” function in the R package “caret” (version 6.0-94) with the parameter “*p* = 0.7”. We utilized a predictor (TMB or HGB level) and two predictors (TMB and HGB level) to fit logistic regression models in the training set, respectively. The two models were then applied to the test set. The area under the receiver operating characteristic (ROC) curve (AUROC) was reported to indicate the predictive performance. In the logistic regression analyses, we used the R function “glm()” to fit the binary model with the parameter “family” = “binomial” and other parameters as default. The R function “predict()” with the parameter “type = response” was used to predict the responsiveness of patients in the test set. The R package “ROCR” (version 1.0-11) was used to calculate the AUROC value and R package “multipleROC” (version 0.1.1) was used to plot the ROC curve. To confirm the reproducibility of the predictions, we set different seed numbers for the “createDataPartition()” function to generate 1,000 different combinations of training and test sets.

### Gene-set enrichment analysis

We utilized the single-sample gene-set enrichment analysis (ssGSEA) method (Hanzelmann et al., 2013) to quantify the enrichment levels of gene sets in tumors. A gene set is a collection of marker genes of biological process, pathway, or phenotypic feature. Based on gene expression profiles, the ssGSEA algorithm evaluates a gene set’s enrichment level in a tumor sample. The “GSVA” R package was used to perform the ssGSEA algorithm. The gene sets analyzed in this study are presented in Supplementary Table S2.

### Quantifying HGB levels, homologous recombination defect (HRD), TMB, intratumor heterogeneity (ITH) and MSI in tumors

We defined the HGB level of a TCGA or Pancancer_2020 tumor sample as the average expression levels of four HGB protein-encoding genes, namely *HBA1*, *HBA2*, *HBB* and *HBD*. We obtained HRD scores representing aneuploidy levels in 9,125 TCGA tumors from the publication by Knijnenburg et al. (2018), which defined HRD scores by the assessment of loss of heterozygosity, large-scale state transitions and the number of telomeric allelic imbalances. TMB was defined as the total count of non-synonymous somatic mutations in the tumor. The ITH level was evaluated by the DITHER algorithm (Li et al., 2021), which measures a tumor’s ITH based on its entropies of somatic mutation and copy number alteration profiles. The MSI scores were evaluated by the MSIsensor algorithm (Niu et al., 2014) to indicate the degrees of MSI in tumors.

### Statistical analysis

We employed the Spearman or Pearson method to evaluate the correlations between two continuous variables. We utilized the χ^2^ test with 2,000 Monte Carlo simulations to compare ICI therapy response rates among different HGB baseline levels. One-tailed Mann-Whitney *U* tests were used to compare two classes of non-normally distributed data, including HGB levels, OS and PFS time, and ORR. The DeLong’s test was employed to compare the AUROC values yielded by different models. We implemented all statistical analyses in the R programming environment (version 4.2.1).

## Results

### Baseline HGB levels are associated with clinical outcomes in cancer patients treated with ICIs in a dose-dependent manner

**FIGURE 1:**
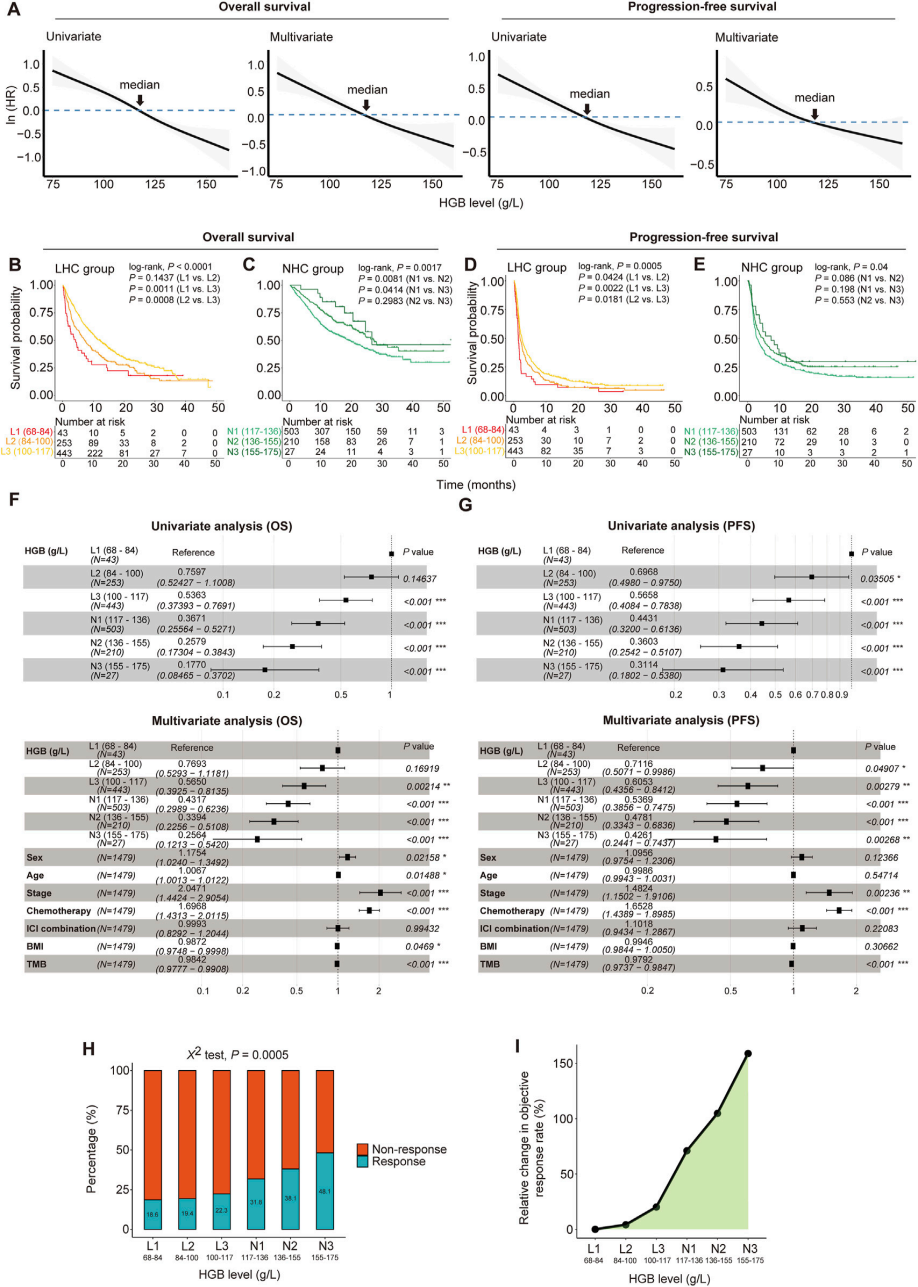
Dose-dependent associations between HGB levels and clinical outcomes in 1, 479 cancer patients treated with immune checkpoint inhibitors (ICIs). **(A)** Hazard ratios (HRs) decrease with HGB levels increasing in patients for both overall survival (OS) and progression-free survival (PFS). The HRs were fitted by spline-based univariate and multivariate cox proportional hazards regression analyses. The multivariate analysis was performed using the covariates of sex, age, BMI, tumor stage, chemotherapy, ICI therapy method and TMB. **(B–E)** The Kaplan-Meier curves to compare survival rates among the subgroups with different HGB levels. Patients were separated into low-HGB-concentration (LHC) group (<117 g/L) and normal-HGB-concentration (NHC) group (≥117 g/L); the LHC group of patients were stratified into three subgroups in the increment of 16 g/L HGB levels, and the NHC group was stratified into three subgroups in the increment of 19 g/L HGB levels. The log-rank test *P*-values are shown. **(F, G)** Forest plots show the associations of HGB levels with OS **(F)** and PFS **(G)** adjusted by other variables. The HRs and 95% CIs were calculated by Cox proportional hazards regression analysis, taking the subgroup with the lowest HGB levels of 68–84 g/L as the reference. **(H)** The ICI therapy response rates of six subgroups of patients stratified by HGB levels. **(I)** The relative changes of the objective response rate (ORR) in the subgroups of patients with different HGB levels, taking the subgroup with the lowest HGB levels (68–84 g/L) as a reference.

Using the spline-based non-linear Cox proportional hazards regression model (Bhaskaran et al., 2018; Lee et al., 2018), we explored the association between pretreatment HGB levels and OS and PFS in 1,479 pan-cancer patients. This analysis predicted the non-linear HR values across the continuous spectrum of HGB levels, taking the median HGB level (117 g/L) as a reference. Univariate Cox proportional hazards regression analysis showed that the HR decreased gradually as the HGB level increased for both OS and PFS (Figure 1A). Furthermore, to explore whether these associations were confounded with other factors, we performed multivariate Cox proportional hazards regression analyses by introducing sex, age, BMI, tumor stage, chemotherapy, ICI therapy method and TMB as covariates. The results showed that the HGB level remained significantly associated with OS and PFS in a dose-dependent manner after adjusting for covariates (Figure 1A).

Based on the cutoff of 117 g/L for HGB levels, we divided the cancer patients into two groups, termed low-HGB-concentration (LHC) group (<117 g/L) and normal-HGB-concentration (NHC) group (≥117 g/L), respectively. We further stratified each group of patients into three subgroups by taking increasements of 16 and 19 g/L HGB levels for LHC and NHC, respectively. Survival analyses showed that the subgroups with higher HGB levels had significantly longer OS and PFS time than those with lower HGB levels (log-rank test, *P* < 0.05) (Figures 1B–E). Furthermore, taking the subgroup with the lowest HGB levels (68–84 g/L) as a reference, we conducted the univariate Cox regression analysis and found a significant positive influence of HGB levels on OS and PFS, as evidenced by that the higher HGB levels, the lower HR values of the subgroups (Figures 1F, G). Again, the similar results were obtained by multivariate Cox regression analyses (Figures 1F, G).

Next, we compared the response rate to ICIs among the six subgroups of cancer patients. Notably, the ORR showed a significant positive correlation with the HGB level (χ^2^ test, *P* = 0.0005) (Figure 1H). Compared with the subgroup with the lowest HGB levels (68–84 g/L), the subgroup with the highest HGB levels (155–175 g/L) achieved an approximately 2.6 times higher ORR (48.1% versus 18.6%) (Figure 1I).

**FIGURE 2:**
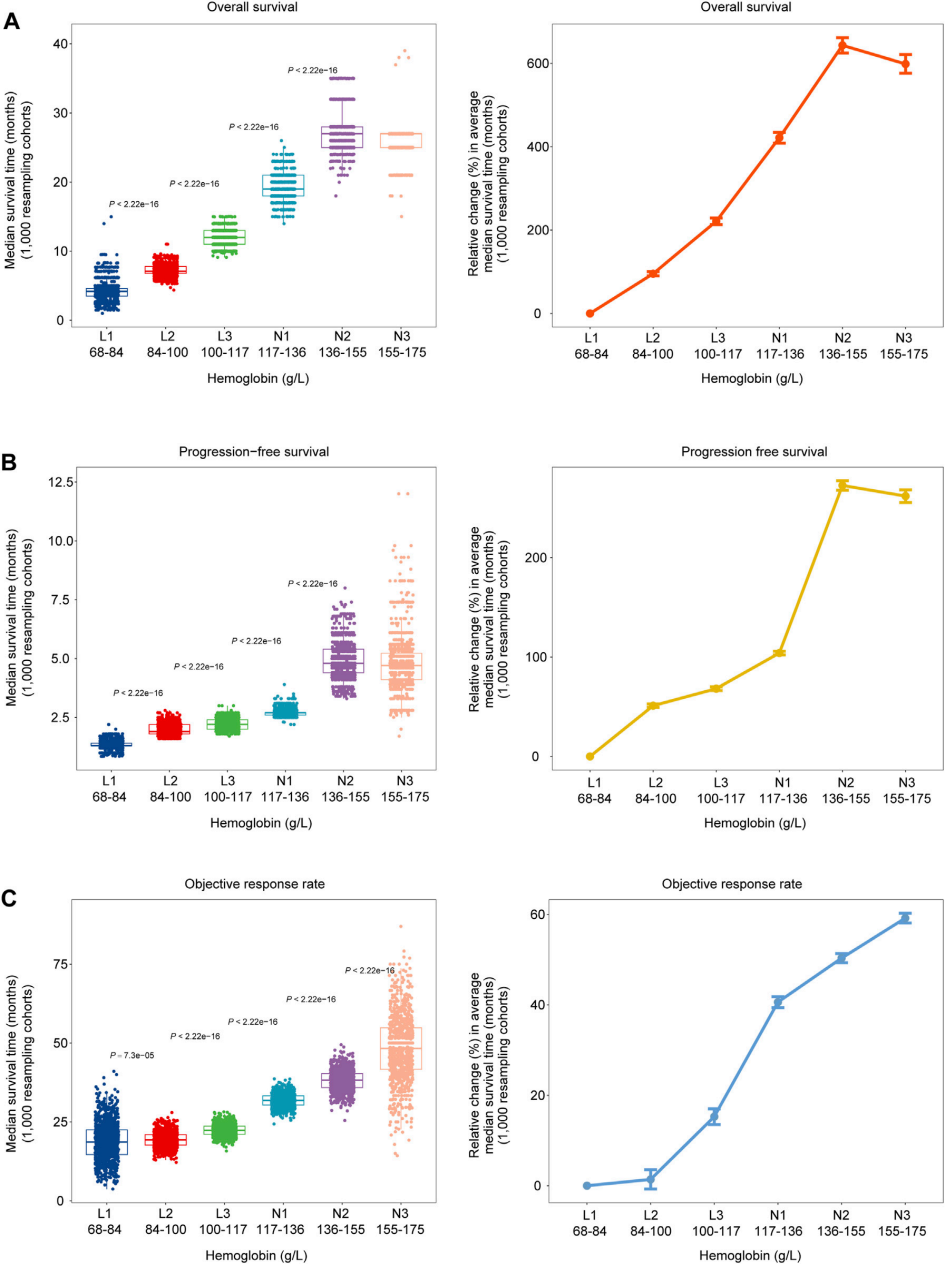
Dose-dependent associations between HGB levels and clinical outcomes in 1,000 bootstrap-resampled cohorts from the 1, 479 cancer patients treated with ICIs. **(A–C)** Changes in median OS time, PFS time and objective response rate (ORR) across six subgroups of patients with different HGB levels (left). Changes in the median OS time, PFS time and ORR across the six subgroups, relative to the subgroup with the lowest HGB levels (68–84 g/L) (right).

To explore the stability and reproducibility of the dose-dependent relationship between HGB levels and ICI therapy outcomes, we analyzed 1,000 cohorts randomly generated from the original 1,479 pan-cancer specimens by the bootstrap resampling method. We observed that the median OS time increased with the HGB level increasing, except that the subgroup (N3) with the highest HGB levels had a slightly shorter median OS time than the subgroup (N2) with the second highest HGB levels (Figure 2A). The similar result was observed for PFS. The median OS time increased from 4.2 months in the L1 subgroup (HGB level: 68–84 g/L) to 25.6 months in N3 (HGB level: 155–175 g/L), and the median PFS time increased from 1.3 months in L1 to 4.8 months in N3 (Figures 2A, B). As expected, we observed noticeable improvements in ORR with the increasing levels of HGB. ORR increased from 18.8% in L1 to 48.4% in N3, with an approximately 157% relative change (Figure 2C).

### Associations between HGB levels and ICI treatment outcomes across different clinicopathological characteristics, treatment programs and cancer types

**FIGURE 3:**
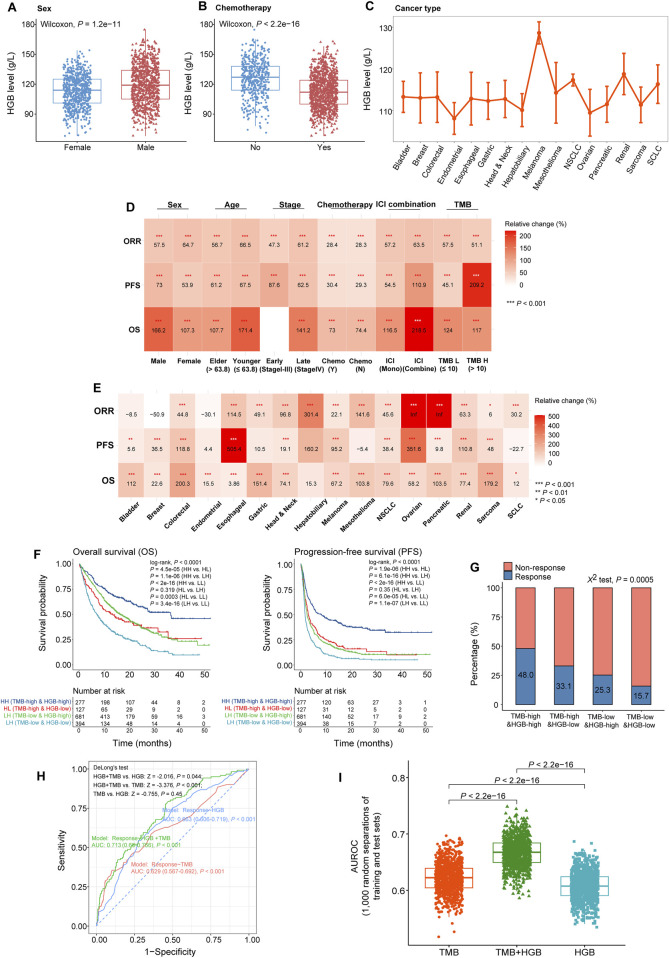
Associations between HGB levels and ICI treatment outcomes across different clinicopathological characteristics, treatment programs and cancer types. **(A, B)** Comparisons of HGB levels between male and female patients **(A)**, and between the patients treated with chemotherapy before ICI therapy and those without chemotherapy **(B)**. **(C)** Comparisons of HGB levels across 16 cancer types. **(D, E)** Heatmap shows the changes of OS, PFS and ORR in the patients with high HGB levels (≥117 g/L) relative to those with low HGB levels (<117 g/L) in the subgroups defined by clinicopathological characteristics **(D)**, treatment program **(D)**, and cancer type **(E)**. Bootstrap was applied to generate 1,000 resampled cohorts for each subgroup **(F, G)**. Kaplan-Meier curves show that the patients with both high TMB and HGB levels have the best OS and PFS **(F)** and the highest ORR **(G)**, while those with both low TMB and HGB levels have the worst OS and PFS **(F)** and the lowest ORR **(G)**. **(H)** ROC plots of the predictive results for ICI therapy response versus non-response using HGB level or TMB solely and combination of TMB and HGB level as features, respectively, by logistic regression models. AUROC values were shown in the plots. **(I)** The AUROC values obtained with HGB level or TMB as the feature are significantly lower than those achieved with both TMB and HGB level as features, in the 1,000 different combinations of training and test sets.

In the cohort of 1,479 pan-cancer patients analyzed, HGB levels varied among the groups with different clinicopathological characteristics, treatment programs and cancer types. For example, HGB levels were significantly higher in male than in female patients (one-tailed Mann-Whitney *U* test, *P* = 1.2 × 10^−11^) (Figure 3A). HGB levels were markedly lower in the patients receiving chemotherapy than those not treated with chemotherapy (*P* < 2.2 × 10^−16^) (Figure 3B), consistent with previous reports (Abdel-Razeq et al., 2020). Furthermore, HGB levels varied across different cancer types; the melanoma patients had the highest HGB levels, while some female-specific cancers had the lowest HGB levels, such as endometrial and ovarian cancers (Figure 3C). To explore whether these factors have an impact on the association between HGB levels and ICI treatment outcomes, we analyzed the subgroups of patients with different clinicopathological characteristics, treatment programs and cancer types individually. Here the clinicopathological characteristics included sex, age, tumor stage and TMB, and the treatment programs included chemotherapy before ICI treatment, ICI monotherapy and ICI-combined therapy. Likewise, we randomly generated 1,000 cohorts for each subgroup of patients using the bootstrap resampling method. For each bootstrap-resampled cohort, the patients were divided into two classes (high versus low HGB levels) based on the median HGB level (117 g/L), and the clinical outcomes (OS, PFS and ORR) were compared between both classes. This analysis demonstrated that all these clinical outcomes were significantly improved in the class with high HGB levels relative to the class with low HGB levels within almost all the subgroups of patients (*P* < 0.05) (Figure 3D). Moreover, in the 16 individual cancer types, HGB levels showed a positive association with at least one of the clinical outcomes (Figure 3E). In nine cancer types (colorectal, esophageal, head and neck, melanoma, non-small cell lung, ovarian, pancreatic, renal, and sarcoma cancers), higher HGB levels were correlated with better clinical outcomes of OS, PFS and ORR. Altogether, these results suggest that the positive association between HGB levels and ICI treatment outcomes is independent of the clinicopathological characteristics, treatment program and cancer types.

Strikingly, even in the patients with low TMB [≤10/mega base (mb)], who are known as insensitive to ICIs (Marabelle et al., 2020), higher HGB levels were correlated with better OS, PFS and ORR (*P* < 0.001) (Figure 3D). Furthermore, based on TMB and HGB level, we divided the 1,479 cancer patients into four groups: TMB-high and HGB-high (TMB > 10/mb and HGB ≥ 110 g/L), TMB-low and HGB-high (TMB ≤ 10/mb and HGB ≥ 110 g/L), TMB-high and HGB-low (TMB > 10/mb and HGB < 110 g/L), and TMB-low and HGB-low (TMB ≤ 10/mb and HGB < 110 g/L). Survival analyses showed that the TMB-high and HGB-high patients had the best OS and PFS, while the TMB-low and HGB-low patients showed the worst OS and PFS (*P* < 0.0001) (Figure 3F). It indicates that the patients with both high TMB and HGB levels respond best to ICIs, and the patients with both low TMB and HGB levels have the lowest response rate to ICIs. This indication was confirmed through comparing ORR among the four groups of patients (χ^2^ test, *P* = 0.0005), as shown in Figure 3G. Furthermore, to explore whether the HGB level may enhance the performance of TMB in predicting immunotherapy response, we used HGB level, TMB, and both TMB and HGB level as features, respectively, to predict the response to ICIs by the logistic regression model. First, we randomly separated the 1,497 cancer samples into a training set and a test set, which involved 70% and 30% samples, respectively. In the test set, the HGB level feature obtained AUROC of 0.663 (95% CI: 0.606–0.719), and the TMB feature achieved AUROC of 0.629 (95% CI: 0.567–0.692), compared to 0.713 (95% CI: 0.66–0.766) obtained by using both TMB and HGB level as features (Figure 3H). Statistical analysis showed the difference in AUROC to be significant between the combination feature and individual features (DeLong’s test, HGB + TMB vs. HGB: Z = −2.016, *P* = 0.044; HGB + TMB vs. TMB: Z = −3.376; *P* < 0.001). Next, we repeated the above prediction experiments 1,000 times through 1,000 random separations of the cancer specimens into a training set and a test set. We observed that the AUROC values obtained with different features showed the following pattern: TMB + HGB > TMB > HGB (one-tailed paired Mann-Whitney *U* test, *P* < 2.2 × 10^−16^) (Figure 3I). Taken together, these results suggest that the combination of TMB and HGB level is a more powerful predictor of immunotherapy response than HGB or TMB alone.

Figure 3D shows that the improvements of ICI treatment outcomes with elevated HGB levels in the patients receiving chemotherapy are comparable to those without such a therapy. Furthermore, although the improvement degrees of OS and PFS with increased HGB levels were lower in female than in male patients, the improvement of ORR was higher in the former (64.7% versus 57.5%) (Figure 3D). In addition, the patients receiving ICI-combined therapy achieved greater improvements of OS, PFS and ORR than those receiving ICI monotherapy with increased HGB levels (Figure 3D).

#### Associations of HGB-related genes expressions with phenotypic and immune-relevant molecular features in the TCGA and Pancancer_2020 cancer cohorts not treated with ICIs

**FIGURE 4:**
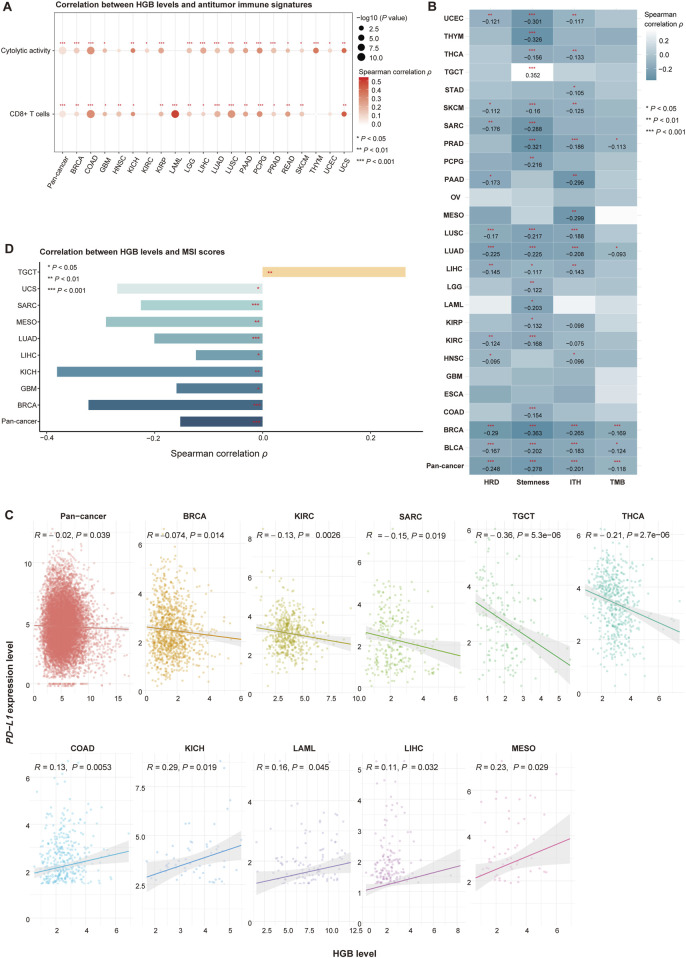
Associations of HGB-related genes expressions with phenotypic and immune-relevant molecular features in the TCGA cancer cohorts not treated with ICIs. **(A)** HGB levels significantly and positively correlate with the enrichment levels of CD8^+^ T cells and cytolytic activity in pan-cancer and in multiple individual cancer types. **(B)** HGB levels significantly and negatively correlate with HRD, stemness and ITH scores and TMB in pan-cancer and in multiple individual cancer types. **(C)** Correlations between HGB levels and *PD-L1* expression levels in pan-cancer and in individual cancer types **(D)** Correlations between HGB levels and MSI scores **(A–D)** only show the cancer types in which the correlations are significant (*P* < 0.05).

To explore potential mechanisms underlying the positive association between HGB levels and the response to ICIs, we analyzed the TCGA and Pancancer_2020 datasets, whose cohorts were not treated with ICIs. Because both cancer cohorts have no HGB-related clinical data available, we quantified a tumor’s HGB level as the average expression levels of four HGB-related genes (*HBA1*, *HBA2*, *HBB* and *HBD*). In the TCGA pan-cancer and 32 individual cancer types, HGB levels showed significant positive correlations with antitumor immune signatures. For example, HGB levels significantly and positively correlated with the enrichment of CD8^+^ T cells in pan-cancer and in 17 individual cancer types, as well as with cytolytic activity in pan-cancer and in 19 individual cancer types (Spearman correlation, *P* < 0.05) (Figure 4A). Similar results were observed in the Pancancer_2020 dataset (Supplementary Figure S1A). These results suggest that high HGB levels are associated with an immune-stimulatory tumor microenvironment in diverse cancers. In addition, previous studies have revealed certain tumor-specific features that promote or correlate with antitumor immunosuppression, such as tumor aneuploidy (Davoli et al., 2017), stemness (Miranda et al., 2019) and ITH (Li et al., 2020b). As expected, HGB levels displayed significant negative correlations with HRD, stemness and ITH scores in pan-cancer and in multiple individual cancer types (*P* < 0.05) (Figure 4B; Supplementary Figure S1B).

Interestingly, although TMB is a positive predictor for immunotherapy response, we did not observe significant positive correlations between HGB levels and TMB in pan-cancer and in individual cancer types (except GBM and ESCA in TCGA and cervical cancer and glioma in Pancancer_2020) (Spearman correlation, *P* < 0.05) (Figure 4B; Supplementary Figure S1B). In contrast, HGB levels showed significant negative correlations with TMB in pan-cancer and in four individual cancer types in TCGA, including BLCA, BRCA, LUAD and PCPG (*P* < 0.05) (Figure 4B), and the significant negative correlation between HGB levels and TMB was also observed in the Pancancer_2020 pan-cancer (Supplementary Figure S1B). These results support the previous finding in the context of immunotherapy that the positive association between HGB levels and immunotherapy response is independent of TMB. PD-L1 expression is another positive predictor for ICI therapy response (Khunger et al., 2017). In TCGA dataset, we observed significant negative correlations between HGB levels and *PD-L1* expression levels in pan-cancer and in five individual cancer types (BRCA, KIRC, SARC, TGCT and THCA) (Pearson correlation, *P* < 0.05) (Figure 4C). In addition, in five cancer types (COAD, KICH, LAML, LIHC and MESO), HGB levels showed significant positive correlations with *PD-L1* expression levels (Pearson correlation, *P* < 0.05) (Figure 4C). Furthermore, in the Pancancer_2020 dataset, HGB levels and *PD-L1* expression levels showed no significant correlation in pan-cancer and in any individual cancer types (Supplementary Figure S1B). These results indicate that the positive association between HGB levels and ICI therapy response is unlikely to correlate with PD-L1 expression. MSI is also a positive predictor for ICI therapy response (Le et al., 2015). In the TCGA cohorts, HGB levels showed significant negative correlations with MSI scores in pan-cancer and in eight individual cancer types, and significant positive correlations in a cancer type (TCGT) (Spearman correlation, *P* < 0.05) (Figure 4D). Again, this analysis demonstrated that the association between HGB levels and ICI therapy response is not related to MSI.

Notably, the HGB level showed no significant correlation with OS and PFS in most of the TCGA cancer cohorts (log-rank test, *P* > 0.05), except that the HGB level had significant positive correlations with OS in LAML and PRAD and significant negative correlations with PFS in MESO (log-rank test, *P* < 0.05) (Supplementary Figure S2). These results are in contrast with the previous finding that HGB levels had significant positive correlations with OS and PFS in the cancer cohorts receiving ICI therapies. It supports that the positive association between HGB levels and clinical outcomes in cancer patients exists exclusively in the background of immunotherapy.

## Discussion

A majority of cancer patients may experience anemia (low HGB level) during disease and/or treatment (Littlewood, 2001). However, the insidious risk of low HGB levels in cancer patients is usually underestimated. Especially, the impact of HGB levels on cancer treatment outcomes remains insufficiently explored. In this study, we have investigated the association between HGB levels and clinical outcomes in pan-cancer patients treated with ICIs and in those not receiving such therapies. We found that HGB levels were positively correlated with clinical outcomes in cancer patients receiving immunotherapy but not in those without immunotherapy. It indicates that the HGB level has a positive association with immunotherapy response. Furthermore, we have demonstrated that the positive association between HGB levels and ICI treatment outcomes is independent of other clinicopathological characteristics (such as sex, age, tumor stage and TMB), treatment program and cancer type. More importantly, the significant association between HGB levels and ICI treatment outcomes appears to be independent of the established biomarkers of immunotherapy response, including TMB, PD-L1 expression and MSI. It suggests the potential clinical value of the HGB level as a novel biomarker of immunotherapy response. Furthermore, we have shown that the combination of TMB and HGB level are better in predicting immunotherapy response than TMB alone. In addition, by analyzing multi-omics data of the TCGA and Pancancer_2020 cancer cohorts, we have shown that HGB levels are positively associated with antitumor immune signatures (CD8^+^ T cells and cytolytic activity) and negatively associated with tumor properties directing antitumor immunosuppression, such as aneuploidy, stemness and ITH. It supports the role of HGB levels as a positive factor for immunotherapy response. Thus, our findings promote the possibility of HGB levels as an inexpensive and easily accessible predictive biomarker of immunotherapy response.

Compared with previous studies, this study has several strengths. First, different from previous studies which analyzed small number of specimens in a single cancer type (Gou et al., 2021; Zhang et al., 2020), we have analyzed thousands of cancer patients from 16 cancer types. Thus, our results and conclusions are more comprehensive and convincing across pan-cancer. Second, instead of dividing patients based on the reference of normal HGB level, we have uncovered the cumulative impact of HGB levels on ICI therapy outcomes. Our analysis reveals a dose-dependent association between HGB levels and ICI therapy outcomes. Finally, we have taken into account the patients’ discrepancies in clinicopathological characteristics, treatment program and cancer type, and proved that the association between HGB levels and ICI treatment outcomes is independent of these factors, particularly those established biomarkers of immunotherapy response. Nevertheless, this study has certain limitations. First of all, in analyzing the TCGA and Pancancer_2020 cohorts, we have used the mean expression levels of *HBA1*, *HBA2*, *HBB* and *HBD* to represent HGB levels of cancer patients, which may not fully reflect blood HGB levels. Second, our findings need to be validated in larger cancer cohorts to explore the potential of HGB levels as a reliable biomarker of immunotherapy response.

## Data Availability

The original contributions presented in the study are included in the article/Supplementary Material, further inquiries can be directed to the corresponding authors.

## Supplementary material

SUPPLEMENTARY FIGURE S1 Associations of HGB-related genes expressions with phenotypic and immune-relevant molecular features in the Pancancer_2020 cohort not treated with ICIs. A Correlations between HGB levels and the enrichment levels of CD8+ T cells and cytolytic activity in pan-cancer and in individual cancer types. B Correlations between HGB levels and stemness, TMB and PD-L1 expression levels in pan-cancer and in individual cancer types.

SUPPLEMENTARY FIGURE S2 HGB level showed no significant correlation with OS and PFS in most of the TCGA cancer cohorts.

## Abbreviations

IC, immune checkpoint inhibitor; HGB, hemoglobin; TMB, tumor mutation burden, MSI: microsatellite instability; HRD, homologous recombination defect; ITH, intratumor heterogeneity; ICB, immune checkpoint blockade; OS, overall survival; PFS, progression-free survival; ORR, objective response rate; ssGSEA, single-sample gene-set enrichment analysis; BMI, body mass index; HR, hazard ratios; CI, confidence intervals; ROC, receiver operating characteristic; AUROC, area under the receiver operating characteristic curve.
