# Supplementary figures and images for: The baseline hemoglobin level is a positive biomarker for immunotherapy response and can improve the predictability of tumor mutation burden for immunotherapy response in cancer
Source: Front Pharmacol. 2024 Oct 2;15:1456833. doi: 10.3389/fphar.2024.1456833 (PMC11480016; doi:10.3389/fphar.2024.1456833)

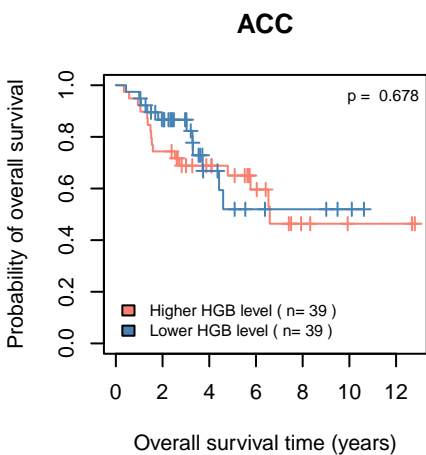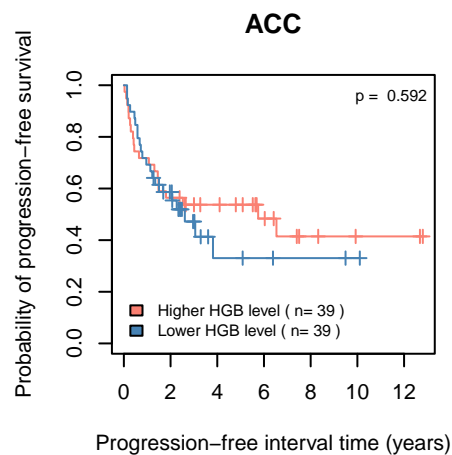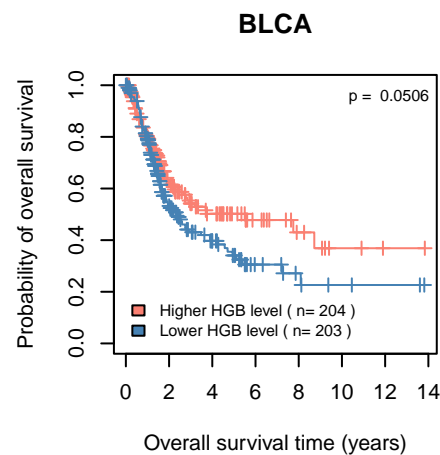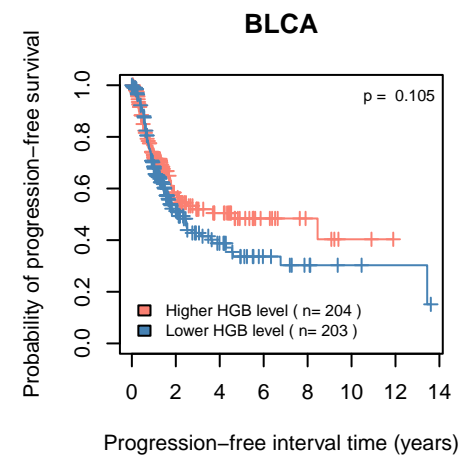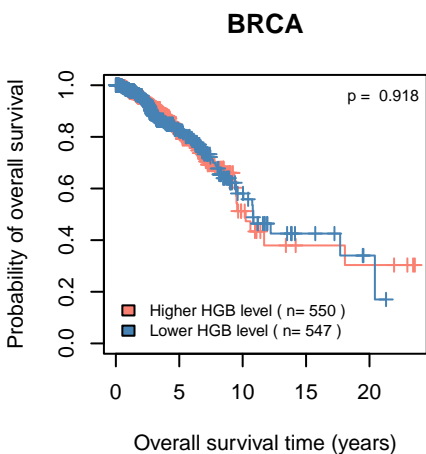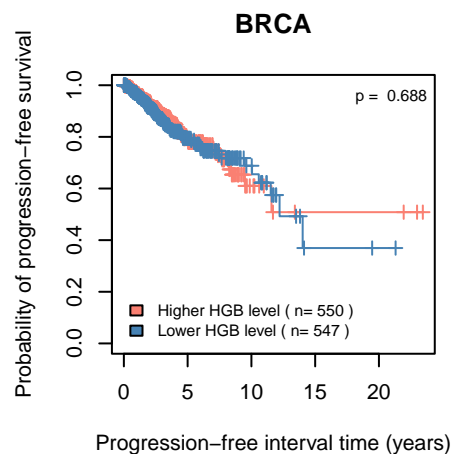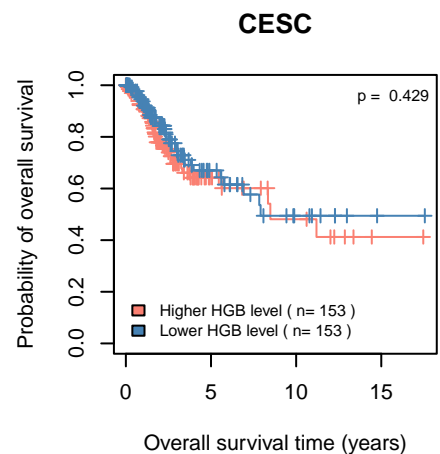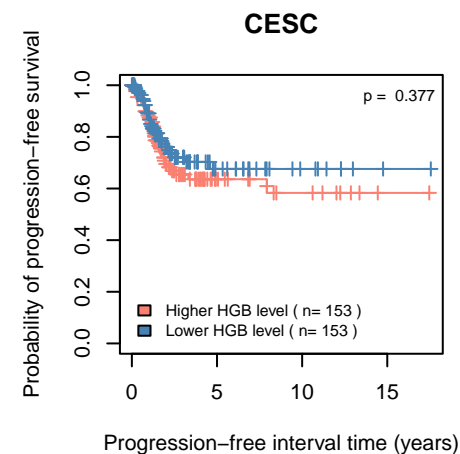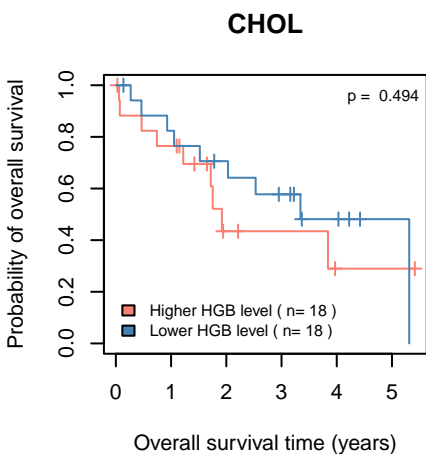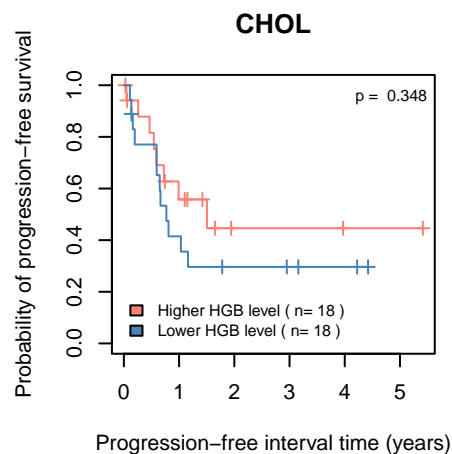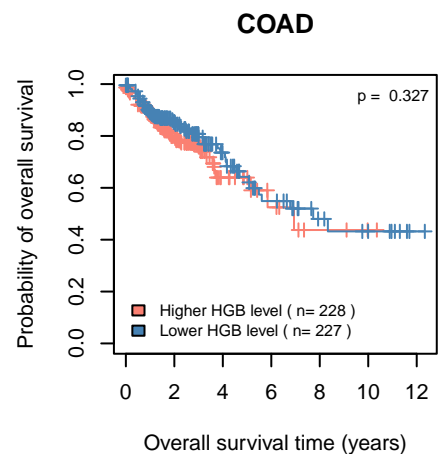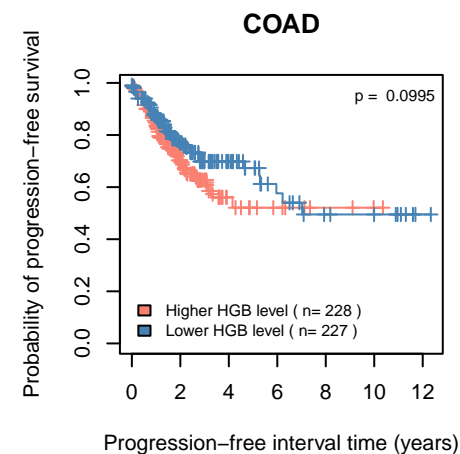

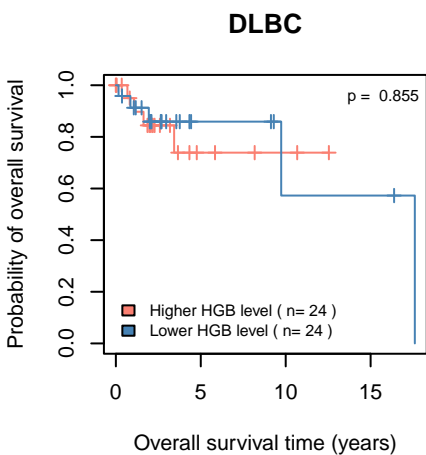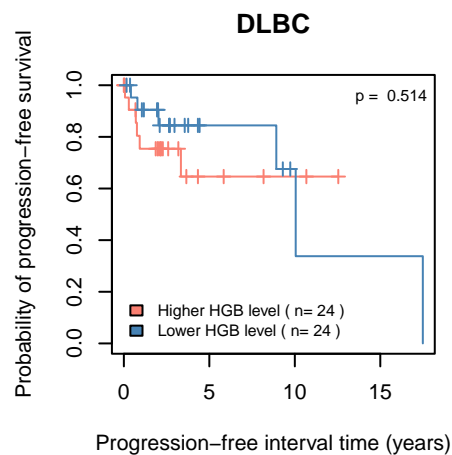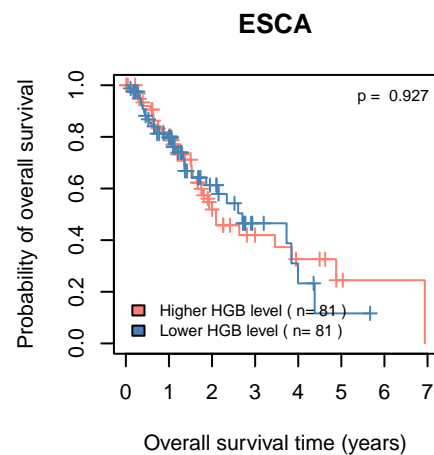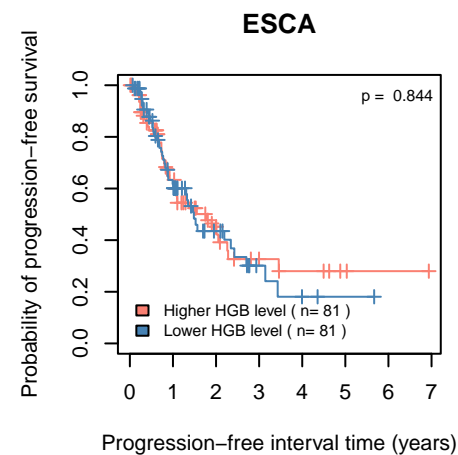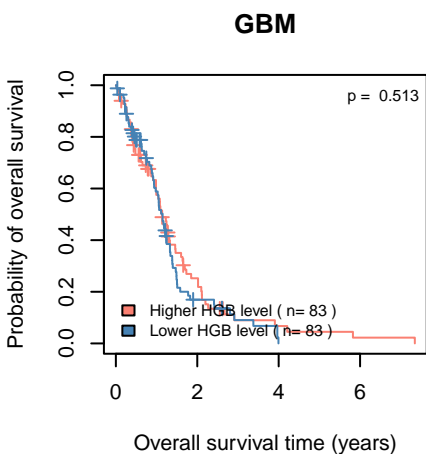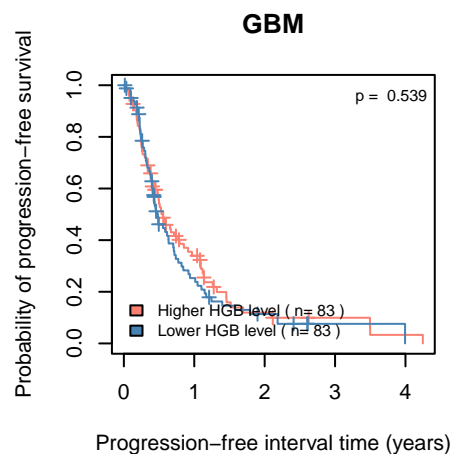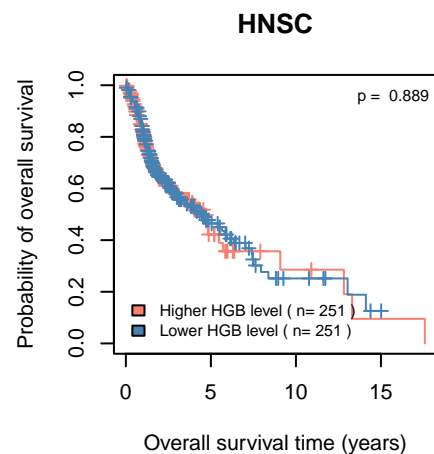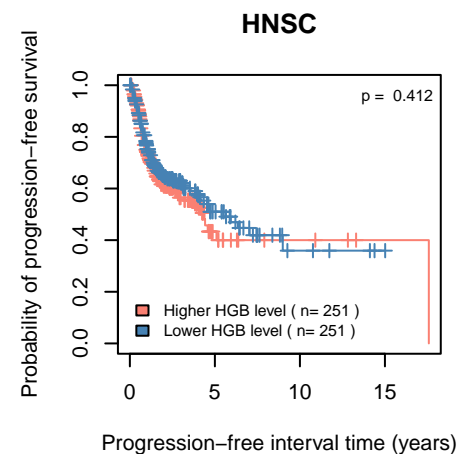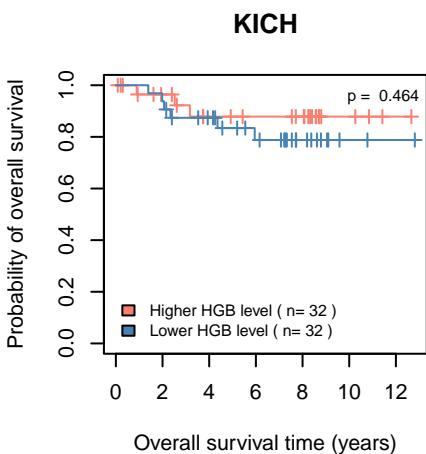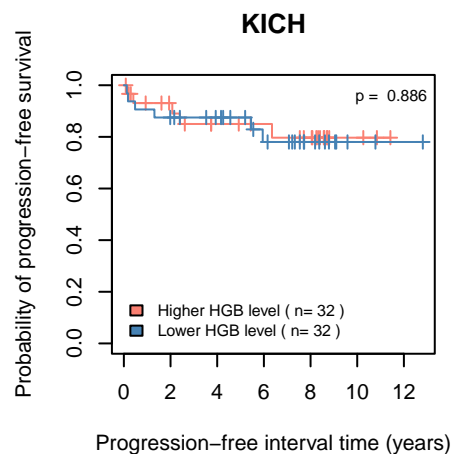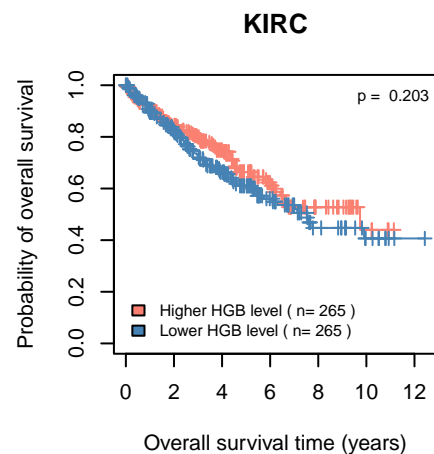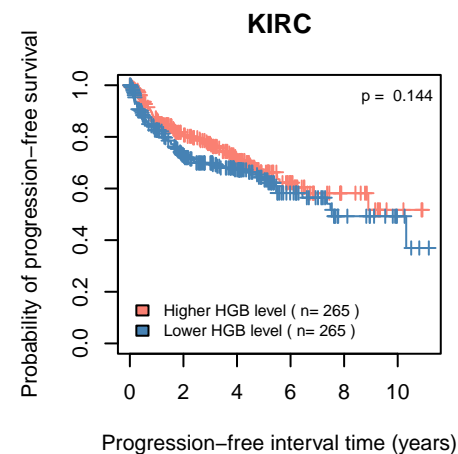

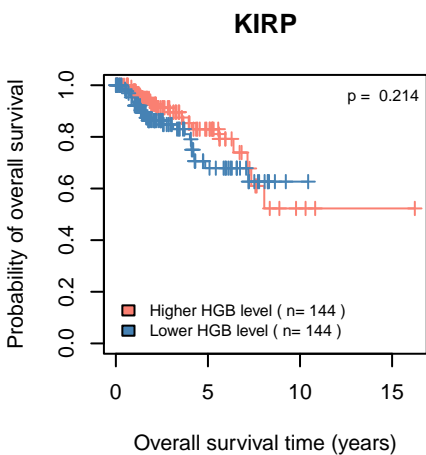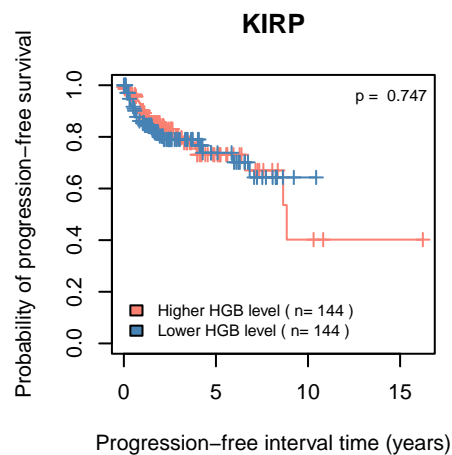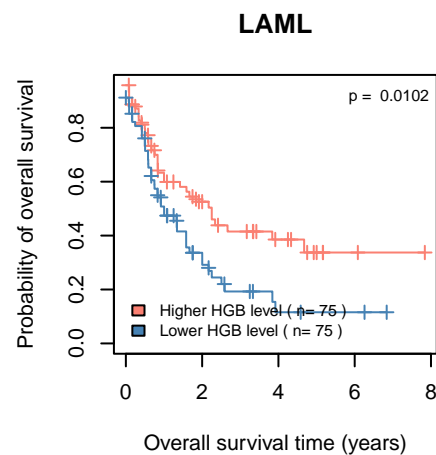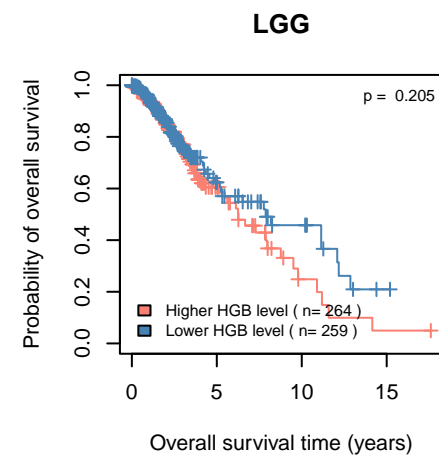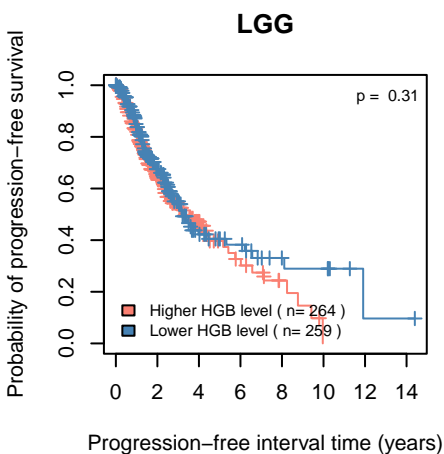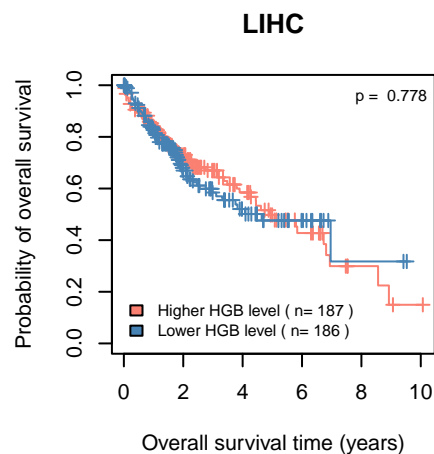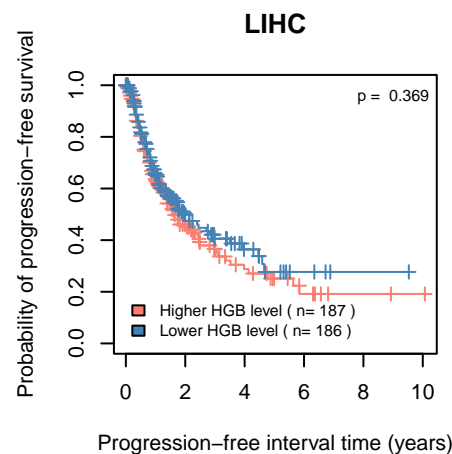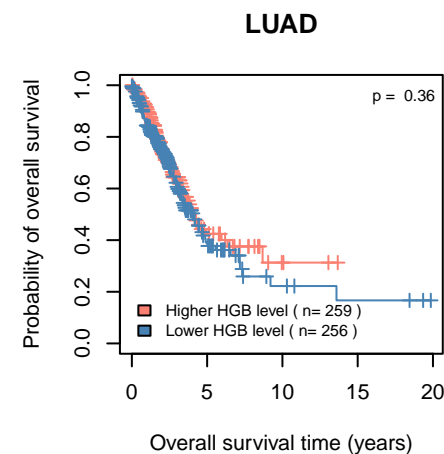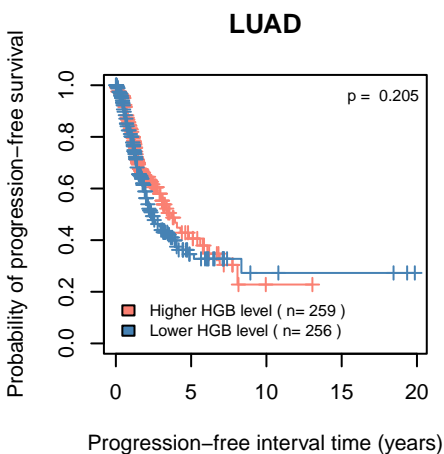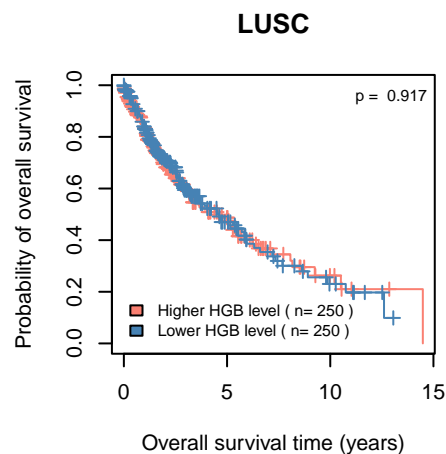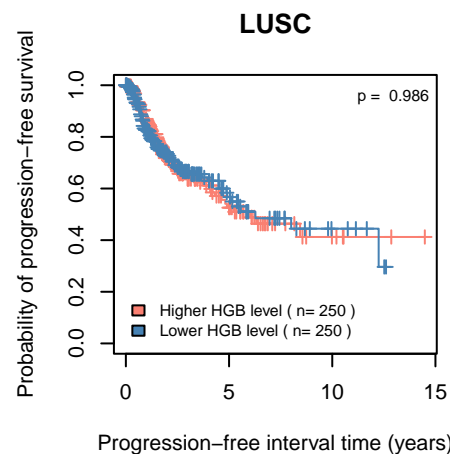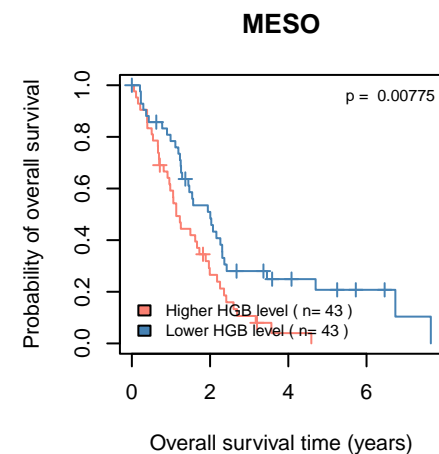

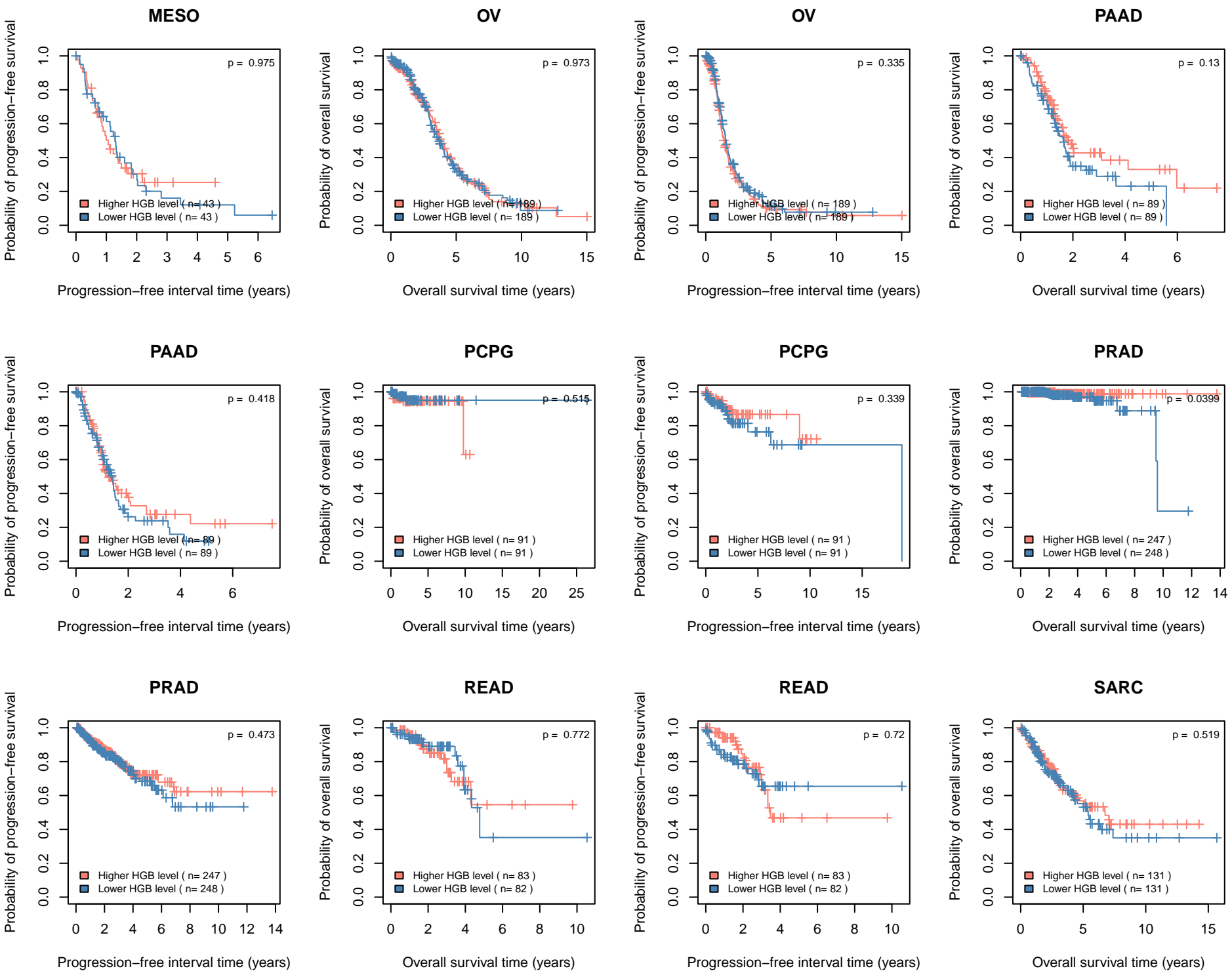

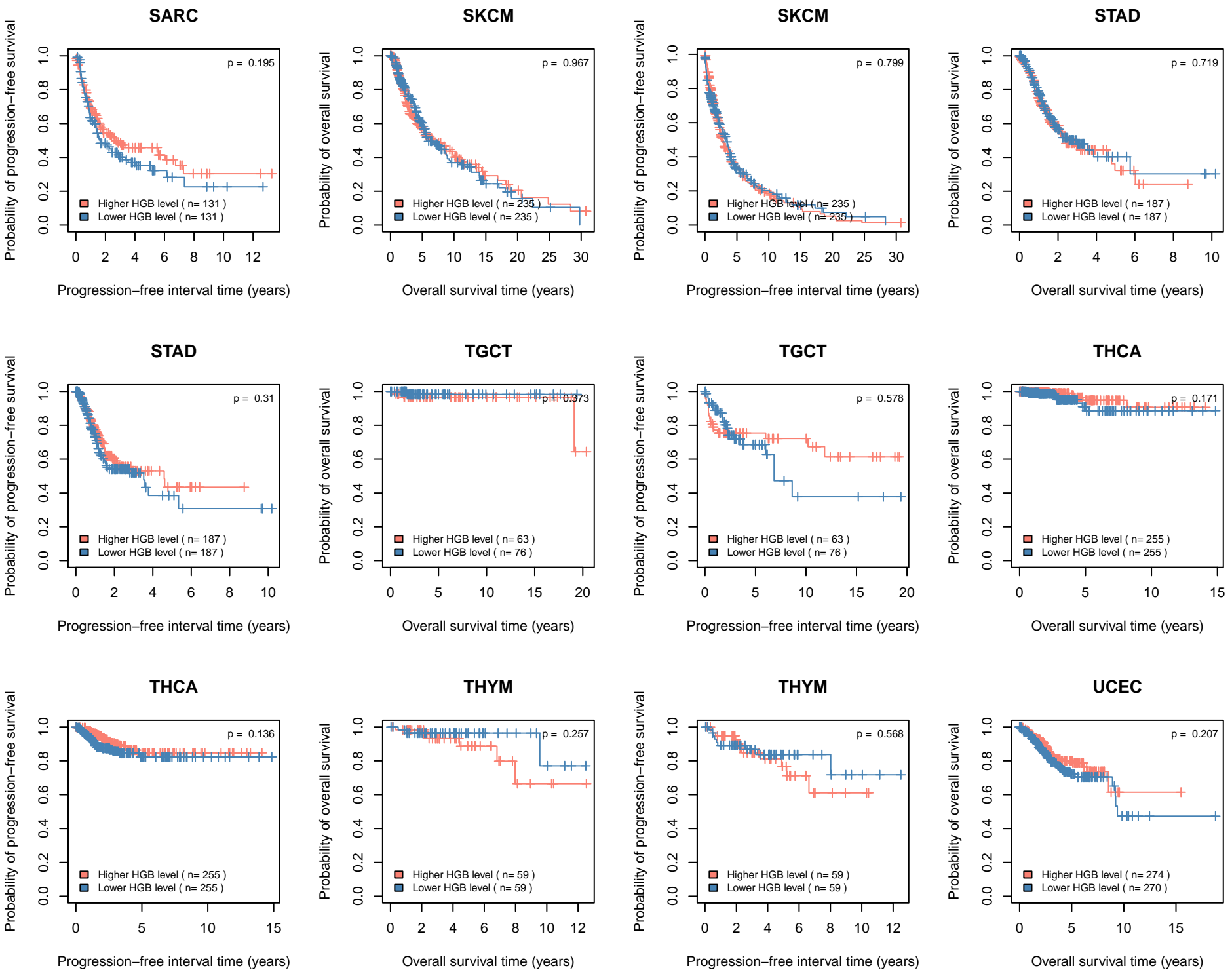

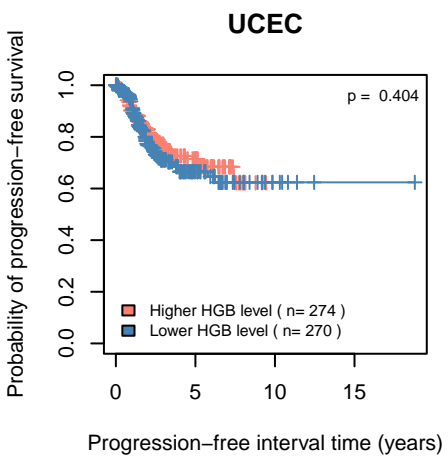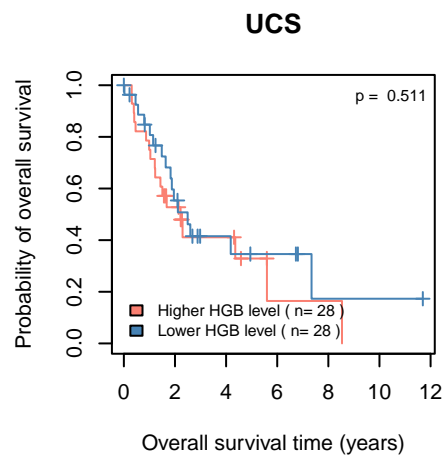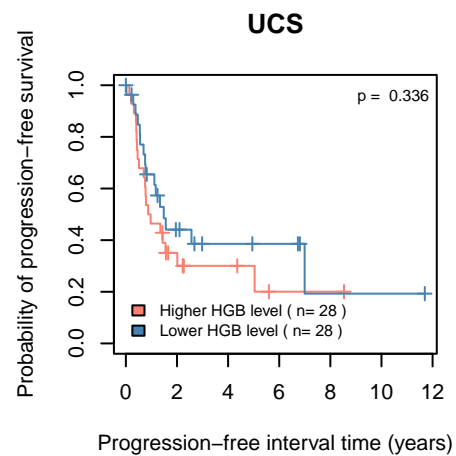

Supplement: Supplementary file 1 [file Image2.PDF]

A

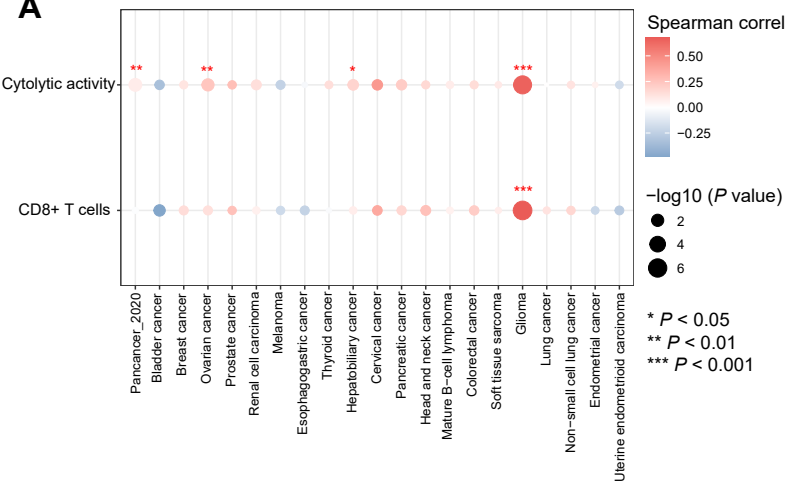

B

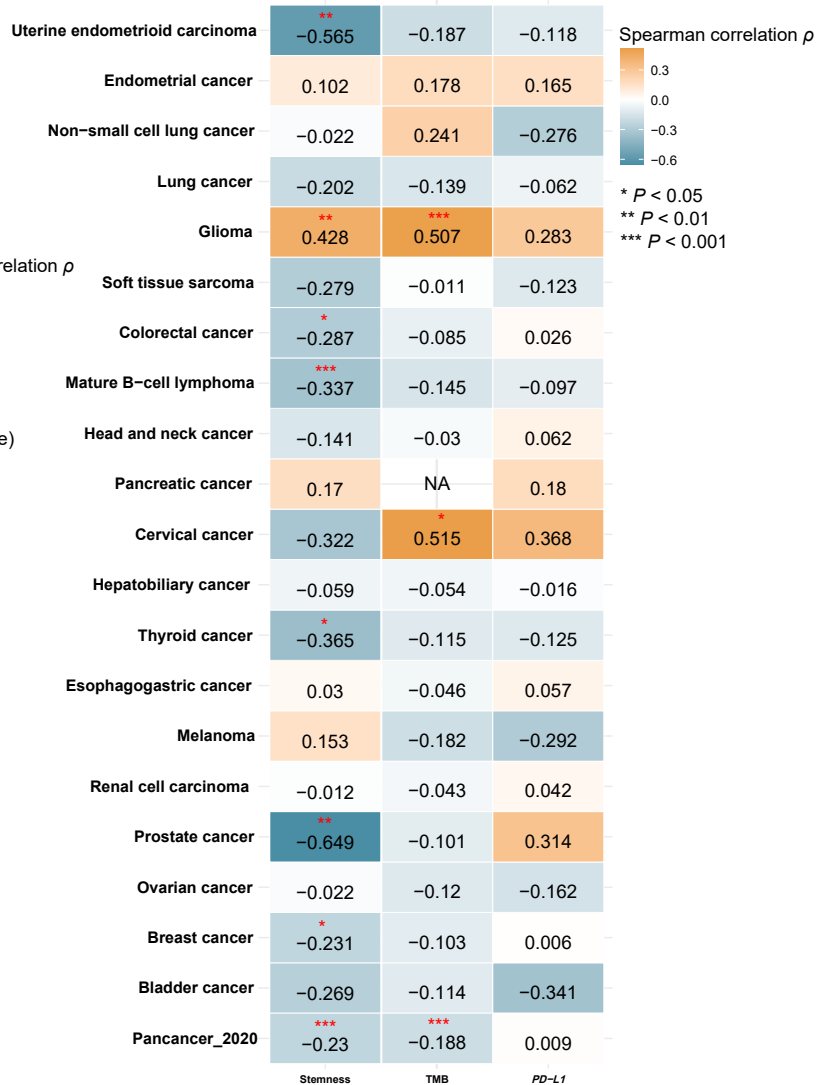

Supplement: Supplementary file 3 [file Image1.PDF]
